# The development of an instrument that can identify children with palliative care needs: the Paediatric Palliative Screening Scale (PaPaS Scale): a qualitative study approach

**DOI:** 10.1186/1472-684X-12-20

**Published:** 2013-05-08

**Authors:** Eva Bergstraesser, Richard D Hain, José L Pereira

**Affiliations:** 1 Department of Palliative Care and Oncology, University Children’s Hospital Zurich, Steinwiesstrasse 75, CH-8032, Zurich, Switzerland; 2 Department of Child Health, University Hospital of Wales, Cardiff Wales, UK; 3 Department of Palliative Medicine and Elisabeth Bruyère Research Institute, Bruyère; Continuing Care and Division of Palliative Care, The Ottawa Hospital and University of Ottawa, Ottawa, Canada

**Keywords:** Palliative care, Children, Family, Assessment, Needs

## Abstract

**Background:**

The introduction of paediatric palliative care and referral to specialised teams still occurs late in the illness trajectory of children with life-limiting diseases. The aim of this ongoing multipart study was to develop a screening instrument for paediatricians that would improve the timely identification of children who could benefit from a palliative care approach.

**Methods:**

We used a qualitative study approach with semi-structured interviews (Part 1) and a focus group discussion (Part 2) to define the domains and items of the screening instrument. Seven international paediatric palliative care experts from the UK, France, USA, and Canada took part in face-to-face interviews, and eleven paediatric health professionals from the University Children’s Hospital, Zurich, participated in a subsequent focus group discussion.

**Results:**

This preliminary phase of development and validation of the instrument revealed five domains relevant to identifying children with life-limiting diseases, who could benefit from palliative care: 1) trajectory of disease and impact on daily activities of the child; 2) expected outcome of disease-directed treatment and burden of treatment; 3) symptom and problem burden; 4) preferences of patient, parents or healthcare professional; and 5) estimated life expectancy. Where palliative care seems to be necessary, it would be introduced in a stepwise or graduated manner.

**Conclusions:**

This study is a preliminary report of the development of an instrument to facilitate timely introduction of palliative care in the illness trajectory of a severely ill child. The instrument demonstrated early validity and was evaluated as being a valuable approach towards effective paediatric palliative care.

## Background

To be effective, paediatric palliative care (PPC) should be integrated into other aspects of paediatrics [1,2]. A number of studies [2-4] have clarified the needs of children and their families once the child’s disease becomes incurable and progressive; they include symptom management, information, relationship with and access to health professionals, and participation in decision-making. PPC can address these; it is typically delivered through a shared care model, in which the PPC team works alongside primary attending professionals [1,5,6]. The effectiveness of this approach has been shown [6,7], but access to PPC is still limited in some first world countries, most notably in Switzerland. Recent reports [8-10] show that, despite WHO guidelines [11], referrals to palliative care are often made late in the trajectory of a life-limiting disease. This is true even in countries like the UK, where availability of palliative care for children is comparatively good [12-14]. Referral patterns remain less than ideal despite the availability of a number of scales designed to improve recognition of the need for palliative care [15-19], perhaps because they are all designed for adults.

Several factors may contribute to this delay. The potential benefits of PPC may be overlooked early in the trajectory of a condition, especially if the likelihood of control or cure is overestimated [20-22]. For some professionals, death is perceived as a failure of care so that they are reluctant to consider its possibility or proximity [23]. Some professionals are concerned about the term ‘palliative care’ itself [24,25], feeling its interpretation by families may be negative. Finally, professionals may feel under pressure from families to offer treatments that are futile because for some families any alternative is equivalent to ‘giving up’ [26].

The effect of these misunderstandings can be exacerbated by the fact that knowledge and understanding of PPC are often inconsistent [22] and that there are few clear criteria of when PPC should be introduced [20,27]. In adults, the focus of instruments for helping prognostication is on estimating life expectancy, and their use often relates specifically to decision-making around end-of-life issues. In children, by contrast, a palliative care approach can have more diverse aims, reducing burdensome treatments in children suffering from advanced cancer, enhancing quality of life through effective symptom control and easing the emotional burden on parents and the family. To achieve these aims, an instrument for children must capture the child’s palliative care needs at an earlier stage than those used in adults [15,16].

We have developed a model for an instrument, called Paediatric Palliative Screening Scale (PaPaS Scale) that is designed to help identify children who would benefit from PPC in order to facilitate timely and appropriate referral.

### Aims

The overall aim of the project is to develop a tool to facilitate appropriate and timely involvement of specialist PPC services in the care of patients. The aims of this initial development phase were: 1) to determine which elements identify children requiring PPC; and 2) to show face and content validity of the items.

## Methods

### Design

**Figure 1:**
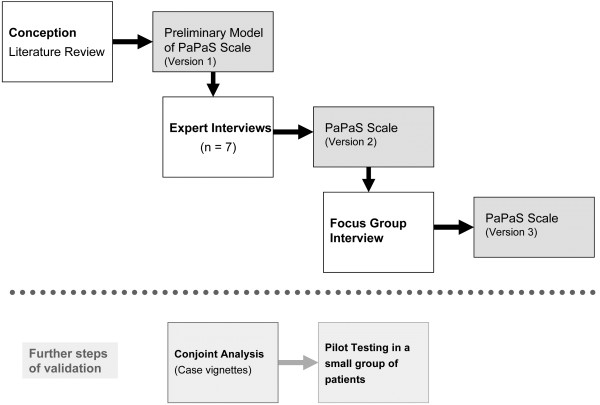
Flowchart of the study.

This study is the initial development phase of a larger project (Figure 1) whose aim is to provide a clinical tool that will facilitate appropriate and timely referral to specialist PPC services. We used an established method [28] that comprises three stages.

1. Development of a conceptual model on the basis of published evidence and the authors’ personal experiences.

2. Description of further items on the basis of expert interviews and a focus group discussion that included providers and users of specialist palliative care.

3. Preliminary testing for face and content validity.

#### Model for the instrument

The basis of the model (Version 1 of the instrument; Table 1) was the initial model, based on published evidence from a literature review on characteristics of children with PPC needs. Neonates and infants (<12 months) were excluded as their disease trajectories and needs are different [29]. The instrument had to accommodate considerable heterogeneity, since PPC encompasses the needs of patients with a wide range of ages (≥1 to ≤19 years) and of diagnoses. The term ‘life-limiting’ is defined in palliative care by the Together for Short Lives Categories (TfSL, formerly ACT [5]; Table 2). The search was extended to include literature on adults as well as on children, from four databases (PubMed, EMBASE, CINHAL, Google Scholar) using the following keywords: ‘paediatric palliative care’, ‘hospice’, ‘assessment’ and ‘needs assessment’ (health service needs and demand). Information was extracted on definitions of PPC, categories of palliative patients, the specific palliative needs of patients and families, prognostic indicators, and illness trajectories. On the basis of these studies, five domains were considered by the investigators (EB, JP) to be relevant to developing the model:

1. Estimated life expectancy

2. Expected outcome of treatment directed at the disease

3. Performance status,

4. Symptom and problem burden

5. Preferences of patient, family or healthcare professional.

**Table 1:** Version 1 of the PaPaS Scale

**Domain and Item number**	**Item**	**Characteristic**	**Score (preliminary)**	**Changes made to Version 1**
**Domain 1**	**Estimated life expectancy**			Position: end of the scale
1.1	Estimated life expectancy	> 2 years	0 □	Qualitative time frames (e.g. weeks to months)
		> 1 but < 2 years	1 □	
		3 months to 1 year	2 □	
		< 3 months	3 □	
1.2	“Would you be surprised if this patient were still alive in 6 months time?”	Yes	3 □	
		No	0 □	
**Domain 2**	**Expected outcome of current treatment directed at the disease and burden of this treatment**			
2.1	Expected outcome of treatment directed at the disease	There are no treatments currently that can cure the disease or prolong life.	4 □	Percentages omitted Outcome of treatment described with respect to life extension and quality of life
		Current treatment patient is receiving or will be receiving may, in only a small number of cases (<20%), prolong life but will not cure.	3 □	
		Current treatment patient is receiving or will be receiving may, in >20% of cases, prolong life but will not cure.	2 □	
		Current treatment patient is receiving or will be receiving may cure the illness in <20% of cases.	1 □	
		Current treatment patient is receiving or will be receiving may cure the illness in >20% of cases.	0 □	
2.2	Burden of treatments	Treatments carry a high level of burden (many side effects).	2 □	Further explication of ‘burden’ (e.g. side effects, stay in hospital)
		Treatments carry a low to medium level of burden (few side effects).	1 □	
		Treatments carry no or minimal burden (side effects) or no treatment is envisioned.	0 □	
**Domain 3**	**Performance status**			Rephrased: ‘Trajectory of disease and impact on daily activities of the child’ Position: beginning of the scale
3.1	Current performance status (in comparison with the child’s own baseline)	Moderate to severe restriction of play (no active play, requires assistance for quiet play) 0-40% of normal range.	3 □	Change of characteristics and omission of percentages
		Mild to moderate restriction of play (able to engage in some active play; requires assistance) 50-70% of normal range.	1 □	
		Normal range of play (able to carry on usual play activities) 80-100% of normal range.	0 □	
3.2	Rate of decline of performance status	Overall, performance has decreased by half over the last 4 weeks.	2 □	Time frame and impact of decline included in 3.1
		Overall, performance has decreased by about a third over the last 4 weeks.	1 □	
		Overall performance has not deteriorated over the last 4 weeks.	0 □	
**Domain 4**	**Symptom and problem burden**			
4.1	Number of symptoms	Patient has 3 or more symptoms (e.g. pain, weight loss, fatigue, dyspnoea, nausea & vomiting, depression, anxiety)	4 □	List of symptoms amended
		Patient has 2 symptoms	3 □	
		Patient has 1 symptom	2 □	
		Patient is asymptomatic	0 □	
4.2	Symptom intensity	Any symptom is severe (equivalent to >6 out of 10)	3 □	Intensity amended by controllability
		Any symptom is moderate (equivalent to 4–6 out of 10)	2 □	
		Any symptom is mild (equivalent to 3 or less out of 10)	1 □	
		Symptoms are absent	0 □	
4.3	Psychological distress of patient	Significant	2 □	
		Mild to moderate	1 □	
		Absent	0 □	
4.4	Psychological distress of parent(s)	Significant	2 □	
		Mild to moderate	1 □	
		Absent	0 □	
4.5	Psychological distress of siblings	Significant	2 □	Omission
		Mild to moderate	1 □	
		Absent	0 □	
**Domain 5**	**Preferences of patient, family and health professional**			
5.1	Request by patient and family	Patient specifically requests a palliative care approach.	4 □ Yes	Omission
			0 □ No	
		Family specifically requests a palliative care approach.	4 □ Yes	
			0 □ No	
5.2	Preference of health professional	You feel that this patient would definitely benefit from a palliative care approach.	4 □ Yes	
			0 □ No	
		**Total score:**		

**Table 2:** **Diagnostic groups qualifying for palliative care (according to Together for Short Lives, formerly ACT)**[5]

**Category**	**Examples**
**Group 1**	
Life-threatening conditions for which curative treatment may be feasible but can fail.	Cancer, heart defects, irreversible organ failures
**Group 2**	
Conditions where premature death is inevitable. Treatment may aim at prolonging life and allowing normal activities.	Cystic fibrosis, Duchenne muscular dystrophy
**Group 3**	
Progressive conditions without curative treatment options. Exclusively palliative treatment may extend over many years.	Metabolic disorders, neuromuscular diseases
**Group 4**	
Irreversible but non-progressive conditions causing severe disabilities leading to susceptibility to health complications and likelihood of premature death.	Severe cerebral palsy

Each domain was further divided into two to five questions (items) for a total of 13 items. Each item provided at least two choices (scored 0–4). Higher individual or total scores indicate a greater need for PPC.

**Figure 2:**
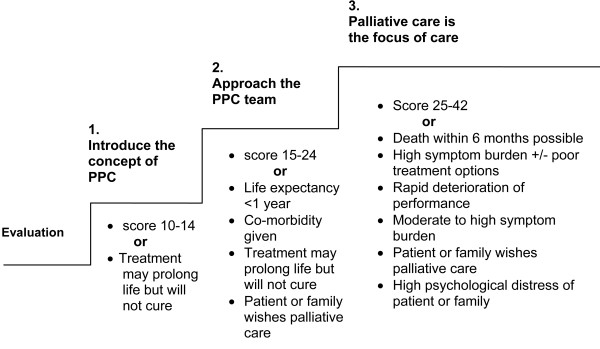
Stepwise approach (Version 1 of the instrument).

In clinical practice, the decision to refer an individual child to palliative care is often made in a way that is graduated, rather than in a single moment, and this is reflected in the model by considering the process conceptually in three steps (Figure 2):

1. *Introduction of Palliative Care* – consider introducing the concept of PPC

2. *Palliative Care Approach* – basic symptom management alongside treatments to control the disease

3. *Palliative Care Focus* – PPC becomes the focus of care.

### Setting/participants

#### Phase 1-expert interviews

The semi-structured interviews with PPC experts included a reflection on the definition of PPC and how they identified children with PPC needs. This was followed by the introduction of the instrument (Version 1; Table 1 and Figure 2) and the invitation to provide preliminary qualitative evaluations of face and content validity [30]. Face validity relies on whether the instrument looks like it measures what it was intended to measure. Content validity describes whether the chosen items of an instrument are representative for the concept of the instrument (in our case intending to measure palliative care needs of children). The opinions of experts are used to evaluate face and content validity in an early stage of instrument development.

The participants were purposively selected specialist practitioners in PPC. Practitioners were invited on the basis of their clinical leadership in their country, region, academic institution or organisation. They included five paediatricians (aged 45–59) and two nurses (ages 47-58yrs) from the United Kingdom, France, the United States and Canada. Before becoming a full-time PPC expert, the participants had worked in oncology, intensive care, neurology and general paediatrics.

The interviews were conducted by the principal investigator (EB) between June and September 2008. They were recorded digitally and transcribed verbatim. The transcripts were coded by hand using qualitative content analysis in which consensus-finding final themes and sub-themes were identified by two investigators (EB, JP). Illustrative quotations were extracted [31]. Data from this first phase were used to modify the instrument (Version 2; Table 3) and as the basis for developing the structured interview for the focus group discussion.

**Table 3:** Version 2 of the PaPaS Scale

**Domain and Item numbers**	**Item**	**Characteristic**	**Score**	**Changes made to Version 2**
**Domain 1**	**Trajectory of disease and impact on daily activities of the child**			
1.1	Trajectory of disease and impact on daily activities of the child (in comparison with the child’s own baseline) (with reference to the last 4 weeks up to a few months)	Stable	0 □	Time frame: 4 weeks, Characteristics: unified, Amended: increase of hospital admissions
		Slowly deteriorating without impact on daily activities.	1 □	
		Unstable with frequent absences from school or restriction of daily activities.	2 □	
		Significant and rapid deterioration with severe restriction of daily activities.	4 □	
**Domain 2**	**Expected outcome of treatment directed at the disease and burden of treatment**			
2.1	Treatment for the disease	… does not cure and has no effect on quality-of-life.	3 □	Meaning of ‘treatment’ outlined further, Characteristics further divided
		… does not cure but has a positive effect on survival and quality-of-life.	2 □	
		… may cure or will prolong survival significantly.	1 □	
2.2	Burden of treatment, (**Burden** means side effects of treatment as well as additional burdens such as stay in hospital)	High level of burden	2 □	
		Low to medium level of burden	1 □	
		No or minimal burden or no treatment is envisioned.	0 □	
**Domain 3**	**Symptom and problem burden**			
3.1	Number of symptoms, (e.g. pain, dyspnoea, nausea/vomiting, weakness/fatigue, anxiety/depression, weight loss, neurological symptoms), (during the last 4 weeks)	≥ 3 symptoms	4 □	Number and listing of symptoms replaced and summarised by intensity and controllability
		2 symptoms	3 □	
		1 symptom	2 □	
		Patient is asymptomatic	0 □	
3.2	Symptom intensity or difficulty of symptom control estimation following VAS scale 0–10, (over the last 4 weeks)	Any symptom is severe (equivalent to ≥6 out of 10)	3 □	
		Any symptom is moderate (equivalent to 4–6 out of 10)	2 □	
		Any symptom is mild (equivalent to ≤ 3 out of 10)	1 □	
		Patient is asymptomatic	0 □	
3.3	Psychological distress of patient due to symptoms	Significant	2 □	Provisional change of scores
		Mild to moderate	1 □	
		Absent	0 □	
3.4	Psychological distress of parents or family due to symptoms	Significant	2 □	
		Mild to moderate	1 □	
		Absent	0 □	
**Domain 4**	**Preferences of health professional**			
4.1	Preference of health care professional	You feel that this patient would definitely benefit from palliative care.	4 □ Yes	Preference of patient and family reintroduced counting only one answer
			0 □ No	
**Domain 5**	**Estimated life expectancy**			
5.1	Estimated life expectancy	Several years	0 □	Change to an either-or-question
		Months to 1–2 years	1 □	
		Weeks to months	2 □	
		Days to weeks	3 □	
5.2	“Would you be surprised if this patient were still alive in 6 months time?”	Yes	3 □	
		No	0 □	
		**Total score:**		

#### Phase 2-focus group discussion

Phase 2 further explored the face and content validity of Version 2 of the instrument with a group of paediatric generalists and sub-specialists from the University Children’s Hospital Zurich. This provided input from potential users of the instrument but who were outside specialist PPC [32]. The instrument was translated into German (EB) to complement the original English version. Eleven participants (4 physicians, 3 nurses, 2 social workers, 1 psychologist, and 1 physiotherapist) were selected purposively and represented a broad spectrum of sub-specialties (cardiology, intensive care, neurology, and oncology). All participants had long professional experience (10-35yrs; median 17yrs) and all included among their patients some whose condition could not be cured. The focus group discussion was facilitated by the principal investigator (EB), and recorded by means of contemporaneous notes made by a research assistant, which were then verified by participants.

The study was approved by the Ethics Review Board (University Children’s Hospital, Zurich). All participants provided written consent.

## Results

### Phase 1

#### General themes

##### Definition of PPC

Participants were supportive of the three most commonly used definitions (World Health Organisation, WHO [11]; Together for Short Lives, formerly ACT [5]; and American Academy of Pediatrics [33]). The definitions were felt to be broad and relatively non-specific. Participants acknowledged that although they were inclusive and accommodated the complexity inherent in PPC, they also lacked precise definition, which potentially made them unsuitable in discussions with healthcare managers or politicians. Furthermore, they do not provide guidance in respect of when to introduce PPC in an individual patient.

I wouldn't change those. […] it means that palliative care begins quite early and goes on quite far. […] when we see the consequences of life-threatening illness on the family it is palliative care.

[…] what it doesn't do all by itself is to dictate how that should be done. It says what palliative care is but it doesn't make some first steps in order to say how that should be worked out practically.

##### When to initiate PPC?

The most appropriate point at which PPC should be introduced in the care of a child was viewed as being individual to the patient, and as depending on a number of factors, including the anticipated prognosis, any life-threatening events within the context of a complex chronic condition, worsening of symptoms, and increasing needs of patients and families. Availability of services locally was also seen to be an influential factor. Most participants underscored the importance of flexibility in considering the optimum time to initiate a discussion with parents about starting PPC.

I also differentiate between kids with complex chronic conditions and those who have a life-threatening component to their complex chronic condition. […] usually the trigger is a life-threatening event or a decision around an intervention of a symptom crisis.

I think when there is a significant risk that the child will not survive. The hard part is, what does that mean? Survive for how long? […] this may not be this hospitalisation it may be the future.

##### Could a screening instrument be useful?

Most participants felt an instrument for facilitating timely and appropriate involvement of PPC would be a valuable tool for improving care of children with life-limiting conditions in clinical practice. Potential benefits identified by participants included screening for appropriate patients, supporting the decision-making process at the end of life, and education of professional colleagues.

[…] it is extremely valuable. […] In practice we need to deal with people who need to know just palliative care, is it necessary or not. […] What you are doing is saying this particular child, irrespective of the diagnosis, is getting to a point where they need it. […] It exactly addresses the difficulty of applying a philosophy in practice.

I see that as an educational opportunity. […] and reframing of what palliative care can offer. […] A tool that enables sort of a better understanding of how a palliative care programme can help would be very, very helpful.

I think it’s a good idea. Because I think often we are asked, you know “when should we call you” or nurses or chaplains are saying it and the team is saying “not yet”, so I think it’s a good thing. […] Some sort of instrument like that would both help teach clinicians what palliative care is, and then perhaps help them think about when to use it […].

#### Instrument-specific themes (version 1)

All participants agreed that the five domains that had been identified were important and appropriate, but there were some specific comments on each. Modifications resulting from the expert interviews are listed in Table 1.

##### Domain 1: estimated life expectancy

This domain provided the most controversy. Participants identified three problems in considering life expectancy:

Lots of people with life expectancy of more than 2 years have a bigger need for palliative care than ones with a lesser expectancy. It’s wrong; there is something wrong there. Alternatively: ‘life-limiting’, ‘life-threatening’, ‘serious risk of death’.

I don’t think it plays a role […], if you have got a child with spinal muscular atrophy that’s kind of very clear that this child is not going to live more than may be two years. […] a child with cerebral palsy – you got no idea.

•It was deemed difficult to do accurately, and, in addition, specific numbers could imply precise prognosis.

•It would be preferable to replace numbers with terms that were more obviously estimated (such as ‘days to weeks’ or ‘weeks to months’).

•There was a difficulty in relating the individual’s life expectancy and the benefit of introducing PPC.

##### Domain 2: expected outcome of current treatment

Participants agreed that this was an important domain. Four main issues were discussed:

‘Current treatments’ is absolutely important. […] What is really good is expected outcome and burden of treatments.

[…] very hard to apply […] it’s never about cure […] it’s always about prolonging life […], the hope of this treatment is to control the disease for as long as possible.

[…] I would like to see ‘expected outcome of disease-directed treatment’ […] and just a limited number of choices: life extension, life extension along with comfort, comfort alone.

•The term ‘cure’ was perceived not to be applicable in many life-limiting conditions, since they are incurable from the outset. The terms ‘life-extension’ and ‘comfort’ were suggested as alternatives.

•The use of percentages to convey the likelihood of cure was considered to be inappropriate since that likelihood could often not be known with precision.

•Integrating a question prompting the physician to consider the balance between good versus harm, and harm versus cure from a quality-of-life perspective was suggested.

•The term ‘burden’ needed clarification, so that users of the instrument understood its holistic scope, including non-physical aspects such as impact on discharge home or to a hospice.

##### Domain 3: performance status

Participants agreed that performance (functional) status was important, yet challenging. A description of the performance status that applies to all children, including those with severe disabilities, was felt to be elusive. To help grade the functional decline, two suggestions were made:

[…] this could be a good way to assess change. Ask about change in frequency of hospitalisation.

[…] you have also given as much impact to change in performance status as you have to their actual performance status, which is very important, because a child who can do virtually nothing may be so precious to his or her parents.

Instead of ‘performance’, the disease progress could be described as: ‘steady, unstable/changing or deteriorating’ – this could also include change in mobility.

•To include sentinel events, such as missing days in the child’s school/education facility or an increase in hospital admissions for symptom control.

•To rephrase ‘performance’ to a more comprehensive term.

##### Domain 4: symptom and problem burden

Participants agreed on this domain. Four main suggestions were made to improve the items.

So distress of sibling […] – most of our doctors wouldn't know. It depends on the family sometimes the siblings are away. […] how do you measure the distress of a sibling who is 5-years old?

•The selection of symptoms was criticised as too limiting.

•Qualitative aspects concerning symptom intensity, such as controllability were suggested.

•The item ‘psychological distress’ (4.3) was unclear regarding to whom it referred (child, family or both).

•Family burden was seen as particularly pertinent; however, siblings’ distress appeared difficult to identify.

##### Domain 5: request for PPC by patient, family, or health professional

This domain generated significant discussion including two contrasting issues.

[…] nobody is going to do that. […] I would say, seriously, in about 10 years, you know, a handful would actively ask. […] families will say “but let’s take a chemo break”.

[…] some health professionals feel they [patients] should benefit [from palliative care] and you have weighted that quite appropriately, in my view highly. I think it is very good.

[…] In my country, it's good to say “do people request palliative care?” […] It is also a way of saying what do they want and how do I - straight at the beginning - try to organise things.

•It was felt to be very unusual for families to request PPC.

•The question could be useful in that it might prompt users to consider PPC.

•A request by a family member should carry significant weight in terms of prompting the team to introduce PPC.

##### Stepwise approach

The idea of a stepwise approach to introducing PPC was supported with some suggestions for modifications.

The first instruction is just to tell the family what palliative care is or introduce the concept of mortality. The second is to make out a plan […] talk about how you are going to involve palliative care, and the third […] there we go, get to the phone and call them.

I think that’s quite good. Because it goes back to this thing about parents starting this question “is my child going to die in childhood?”.

**Figure 3:**
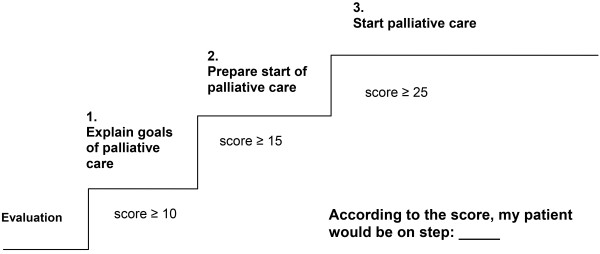
Stepwise approach (Version 2 of the instrument).

Modification: The steps were rephrased (Figure 3).

##### Procedural issues of the instrument

Some concern was expressed that inexperienced clinicians might use it inappropriately. Thus, two interviewees highlighted the possibility of non-referral because of a low score, and another emphasised that the most important aspect about PPC would be to get in touch with the team involved regardless of criteria or scores.

[…] even if the score is less than 25 there are some situations in which you should nevertheless refer.

[…] if we were allowed to introduce what we do with families, we would even have more consults. […] They don't have to make the referral but call to discuss. […] you are not going to bring up death […] you are going to support families […] you are really going to enhance both the team’s and the family’s coping and decision-making.

### Phase 2

#### Instrument-specific themes (version 2)

In general, the concept of the instrument and the stepwise approach were supported, and its clinical usefulness was highlighted.

##### Domain 1: trajectory of disease and impact on daily activities of the child

Participants working with children with neurological conditions highlighted the caveat that some children compensate well for milder deteriorations but decompensate precipitously and disproportionately at a certain point. Thus, a ‘rapid deterioration’ did not seem to apply to many of these children. It was also emphasised that the referring time frame should be clearer.

##### Domain 2: expected outcome of treatment directed at the disease and burden of treatment

Further dividing the characteristics of treatment and burden of treatment, and also clarifying that treatment does not mean disease-related complications such as pain or dyspnoea were suggested.

##### Domain 3: symptom and problem burden

Some participants emphasised the difficulty of symptom assessment in children and suggested eliminating it altogether. There was consensus that the number of symptoms was less relevant than the intensity and impact of the symptom(s). For example, a child with heart failure or under chemotherapy could experience ≥3 symptoms with or without impact on daily living. In addition, psychological distress caused by uncontrolled symptoms was thought to be more important.

##### Domain 4: preference of health professionals

In contrast to Phase 1, participants supported the inclusion of the parents’ request for PPC. Consistent with Phase 1, the misperceptions and misunderstandings about PPC on the part of both professionals and families were highlighted.

##### Domain 5: estimated life expectancy

The majority of participants deemed this element very important. However, it was felt that ‘life expectancy’ and the ‘surprise-question’ represented the same entity and should therefore be aggregated into one single item.

These findings and modifications resulted in Version 3 of the instrument (Table 4).

**Table 4:** Version 3 of the PaPaS Scale

**Domain and Item numbers**	**Item**	**Characteristic**	**Score**
**Domain 1**	**Trajectory of disease and impact on daily activities of the child**		
1.1	Trajectory of disease and impact on daily activities of the child (in comparison with the child’s own baseline) (with reference to the last 4 weeks)	Stable	0 □
		Slowly deteriorating without impact on daily activities.	1 □
		Unstable With impact on and restriction of daily activities.	2 □
		Significant deterioration with severe restriction of daily activities.	4 □
1.2	Increase of hospital admissions, (> 50% within 3 months, compared to previous periods)	No	0 □
		Yes	3 □
**Domain 2**	**Expected outcome of treatment directed at the disease and burden of treatment**		
2.1	Treatment directed at the disease, (does not mean treatment of disease related complications, such as pain, dyspnoea or fatigue)	…is curative.	0 □
		…controls disease and prolongs life with good quality of life.	1 □
		…does not cure or control but has a positive effect on quality of life.	2 □
		…does not control and has no effect on quality of life.	4 □
2.2	Burden of treatment, (**Burden** means side effects of treatment and additional burdens such as stay in hospital in the patient’s or family’s view)	No or minimal burden or no treatment is envisioned.	0 □
		Low level of burden	1 □
		Medium level of burden	2 □
		High level of burden	4 □
**Domain 3**	**Symptom and problem burden**		
3.1	Symptom intensity or difficulty of symptom control (over the last 4 weeks)	Patient is asymptomatic	0 □
		Symptom(s) are mild and easy to control	1 □
		Any symptom is moderate and controllable	2 □
		Any symptom is severe or difficult to control (unplanned hospitalisation or outpatient visits, symptom crises)	4 □
3.2	Psychological distress of patient related to symptoms	Absent	0 □
		Mild	1 □
		Moderate	2 □
		Significant	4 □
3.3	Psychological distress of parents or family related to symptoms and suffering of the child	Absent	0 □
		Mild	1 □
		Moderate	2 □
		Significant	4 □
**Domain 4**	**Preferences/needs of patient or parents**		
	**Preferences of health professional**		
4.1	Patient/parents wish to receive palliative care or formulate needs that are best met by palliative care.	No	0 □ please answer 4.2
		Yes	4 □ do not answer 4.2
4.2	You/your team feel that this patient would benefit from palliative care.	No	0 □
		Yes	4 □
**Domain 5**	**Estimated life expectancy**		
5.1	Estimated life expectancy	Several years	0 □ please answer 5.2
		Months to 1–2 years	1 □ please answer 5.2
		Weeks to months	3 □ do not answer 5.2
		Days to weeks	4 □ do not answer 5.2
5.2	“Would you be surprised if this child were to suddenly die in 6 months time?”	Yes	0 □
		No	2 □
		**Total score:**	

##### Procedural issues of the instrument

Similarly to Phase 1, uncertainty was expressed about who would use the instrument (physician, parents or team), and who would decide about further consequences.

## Discussion

The need to initiate PPC in a timely fashion through improved identification of children and adolescents who might benefit has been highlighted [20,21]. This study establishes early face and content validity for a new instrument to facilitate this identification in clinical practice.

The PaPaS Scale builds on the taxonomy of Together for Short Lives, formerly ACT [5] and underscores that PPC is not limited to end-of-life care. Neonates and infants were excluded as their disease trajectories are mostly short, two thirds of them dying during the first weeks of life [29,34] on the neonatal intensive care unit without episodes of being in their ‘natural’ environment, the family’s home. Even if neonates constitute an important and numerically large proportion of children who could benefit from PPC, they have different needs and we decided not to include them.

The indicators proposed in our instrument operationalize in more detail the approach suggested by Rushton et al. [6]. These authors recommended triggers to identify patients for PPC-conferencing, including: 1) limited life span; 2) ‘surprise-question’ – sudden death within 6–12 months; 3) increase in hospitalisations during the past 6–12 months; 4) major clinical events; 5) symptoms that have changed the frequency of clinic visits; 6) change in response to treatment; 7) conflicts about goals of care. In our experts’ interviews three of these triggers (prognosis, events and symptoms) plus increasing needs of the child and its family were brought up in the first part of the interview, which included a spontaneous reflection on how they identify children with PPC needs. However, with respect to life expectancy (prognosis), the discussions were highly controversial. While several experts of Phase 1 suggested omitting this domain, participants of Phase 2 strongly voted to keep it as it seemed important that this question receives more attention in decision-making. This controversy is interesting; it may reflect cultural differences or the various stages of national PPC achievements, - particularly in Switzerland where PPC is only starting to develop and to be recognized. Thus, in Switzerland, life expectancy may play a stronger role in decision-making towards the introduction of palliative care as compared to UK, the US or Canada where PPC starts to be integrated into care earlier in the course of a disease [20,27].

Predictive factors and events focusing on the diagnosis of dying have also been evaluated by others [34,35]. Brook and Hain [35] proposed that the following candidate factors be included in future studies: frequency of hospital or intensive care admission, episodes of acute illness without recovery to the child’s usual best level of health and physiological changes such as decreased oral intake. Feudtner et al. developed a paediatric mortality prediction model to analyse the likelihood of death during hospitalisation or 1-year post discharge [34]. Among several predictors, the frequency of hospitalisations (>3) during the year before the index hospitalisation and the risk of death were strongly associated. Increase of hospitalisations was also emphasised by some experts from our study and was therefore included in Version 3 of the instrument.

The suggestion by some participants to exclude the domain on preferences of the patient and the family was surprising. This may reflect the difficulties that clinicians (and parents) experience in halting treatment that is futile from a medical perspective. A recent study [36], for example, found that more than a third of children with cancer continued to receive cancer-directed treatment after the parents had realised that there was no realistic chance of cure. However, parents who felt that their child had suffered due to cancer treatment prior to death were particularly unlikely to recommend such a treatment to other families. Our instrument could facilitate such a discussion by a broader exploration of the child’s situation, suffering and needs.

Some reservations concerning the instrument were expressed. There was scepticism about the extent to which clinicians would use the instrument. The challenges in assessing presence and severity of symptoms in children, or estimating life expectancy were highlighted. Written instructions and appropriate training on how to use the instrument will solve some of these concerns. Notwithstanding these, it was felt that the instrument could be a useful clinical and educational tool, increasing earlier activation of PPC and heightening awareness of palliative care needs amongst health professionals. The educational aspect of the tool was particularly seen in the fact that the use of the instrument would evoke discussions which may clarify what palliative care can add in the care of an individual child and its family, and thus translate the definition of palliative care into daily clinical work with severely ill children. Our study suggests a potential role for developing a scoring system linked to this stepwise approach. The predictive validity and clinical effectiveness (impact on children’s quality of life) of such a system will need to be shown.

There are several limitations. This is only the first phase in the development of a new instrument. The interviewer (EB) was also the person who developed the model and analysed the data, thereby potentially introducing bias. The discussions with the supervisor (JP) during the development of the instrument’s model and following the interviews and focus group for checking final themes and sub-themes of data analysis should have reduced the impact of this. The focus group included only clinicians from a German-speaking Swiss hospital, which affects the generalizability of the results. However, testing in different settings and countries is planned. In addition, the perspective of affected families, particularly parents, has not been incorporated yet. This is planned for the following steps of the instrument’s validation.

## Conclusions

The challenges of activating PPC earlier in the illness trajectories of paediatric patients are highlighted. The PaPaS Scale is a new instrument that shows some early promise as an aid in identifying children and adolescents who could benefit from PPC, without undermining treatments to control the disease. Obviously, the results of this study still represent preliminary work.

### Future research

We plan additional psychometric testing; the next step includes a series of case vignettes to further test the items of the instrument, which will be followed by testing with real patients (Figure 1). The educational role of the instrument in terms of raising awareness of PPC needs in children will also be evaluated in a later phase.

## Competing interest

The authors declare that there is no conflict of interest.

## Authors’ contributions

EB conceived of the study and developed the design of the study together with JP. All interviews were conducted and analysed by EB. JP took part in the analysis and interpretation of the results. RH participated in the design. JP and RH helped to draft the manuscript. All authors read and approved the final manuscript.

## Pre-publication history

The pre-publication history for this paper can be accessed here:

http://www.biomedcentral.com/1472-684X/12/20/prepub

## Reporting issues

The first author had full access to all audio taped data in the study and takes responsibility for the integrity of the data and the accuracy of the data analysis.

This article was the subject of the first author’s Master’s Dissertation to achieve the Master of Science in Palliative Medicine degree at the University of Cardiff, Wales.
